# Author Correction: Seasonal recurrence and modular assembly of an Arctic pelagic marine microbiome

**DOI:** 10.1038/s41467-025-57128-7

**Published:** 2025-02-18

**Authors:** Taylor Priest, Ellen Oldenburg, Ovidiu Popa, Bledina Dede, Katja Metfies, Wilken-Jon von Appen, Sinhué Torres-Valdés, Christina Bienhold, Bernhard M. Fuchs, Rudolf Amann, Antje Boetius, Matthias Wietz

**Affiliations:** 1 Max Planck Institute for Marine Microbiology, Bremen, Germany; 2 Institute of Microbiology, ETH Zurich, Zurich, Switzerland; 3 Institute for Quantitative and Theoretical Biology, Heinrich Heine University Düsseldorf, Düsseldorf, Germany; 4 Ecologie Systématique Evolution, CNRS, Université Paris-Saclay, AgroParisTech, Gif-sur-Yvette, France; 5 Alfred Wegener Institute Helmholtz Centre for Polar and Marine Research, Bremerhaven, Germany; 6 MARUM – Center for Marine Environmental Sciences, University of Bremen, Bremen, Germany; 7 Institute for Chemistry and Biology of the Marine Environment, University of Oldenburg, Oldenburg, Germany

**Keywords:** Water microbiology, Ecological networks, Microbial biooceanography, Ecosystem ecology, Element cycles

Correction to: *Nature Communications* 10.1038/s41467-025-56203-3, published online 3 February 2025

In the version of the article initially published, in Fig. 4c, the “M5 (autumn)” graph was originally a duplicate of the “M4 (summer)” graph and has now been corrected in the HTML and PDF versions of the article.

