# Supplementary material for: Seasonal recurrence and modular assembly of an Arctic pelagic marine microbiome
Source: Nat Commun. 2025 Feb 3;16:1326. doi: 10.1038/s41467-025-56203-3 (PMC11790911; doi:10.1038/s41467-025-56203-3)
Supplement: Supplementary file 1 — Supplementary Information [file 41467_2025_56203_MOESM1_ESM.pdf]

Seasonal recurrence and modular assembly of an Arctic pelagic marine microbiome

Taylor Priest & Ellen Oldenburg, Ovidiu Popa, Bledina Dede, Katja Metfies, Wilken-Jon von Appen, Sinhué Torres-Valdés, Christina Bienhold, Bernhard M. Fuchs, Rudolf Amann, Antje Boetius, Matthias Wietz

Supplementary figures

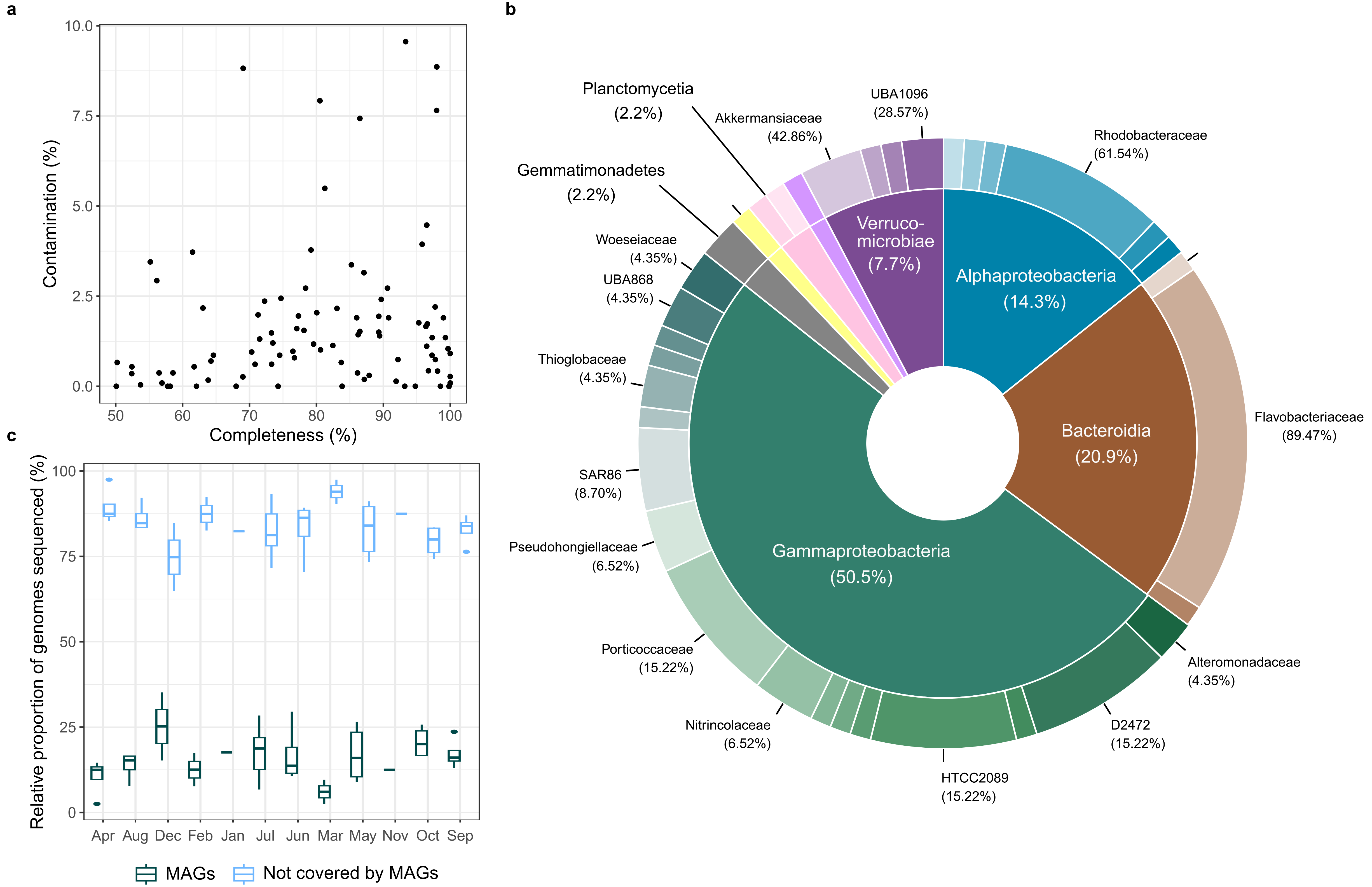

Supplementary Figure 1. Composition and abundance of Metagenome-Assembled Genomes. Metagenome-Assembled Genoms (MAGs) were generated from individual assemblies of each of the 47 PacBio HiFi read metagenomes. **a)** MAGs were clustered into species-level groups, based on 95% ANI, resulting in 91 representatives with completeness >50% and contamination <10%. **b)** The taxonomic composition of species-representative MAGs. **c)** Relative proportion of sequenced genomes captured by the MAGs across sampling months, determined based on average depth of coverage of four ribosomal protein genes.

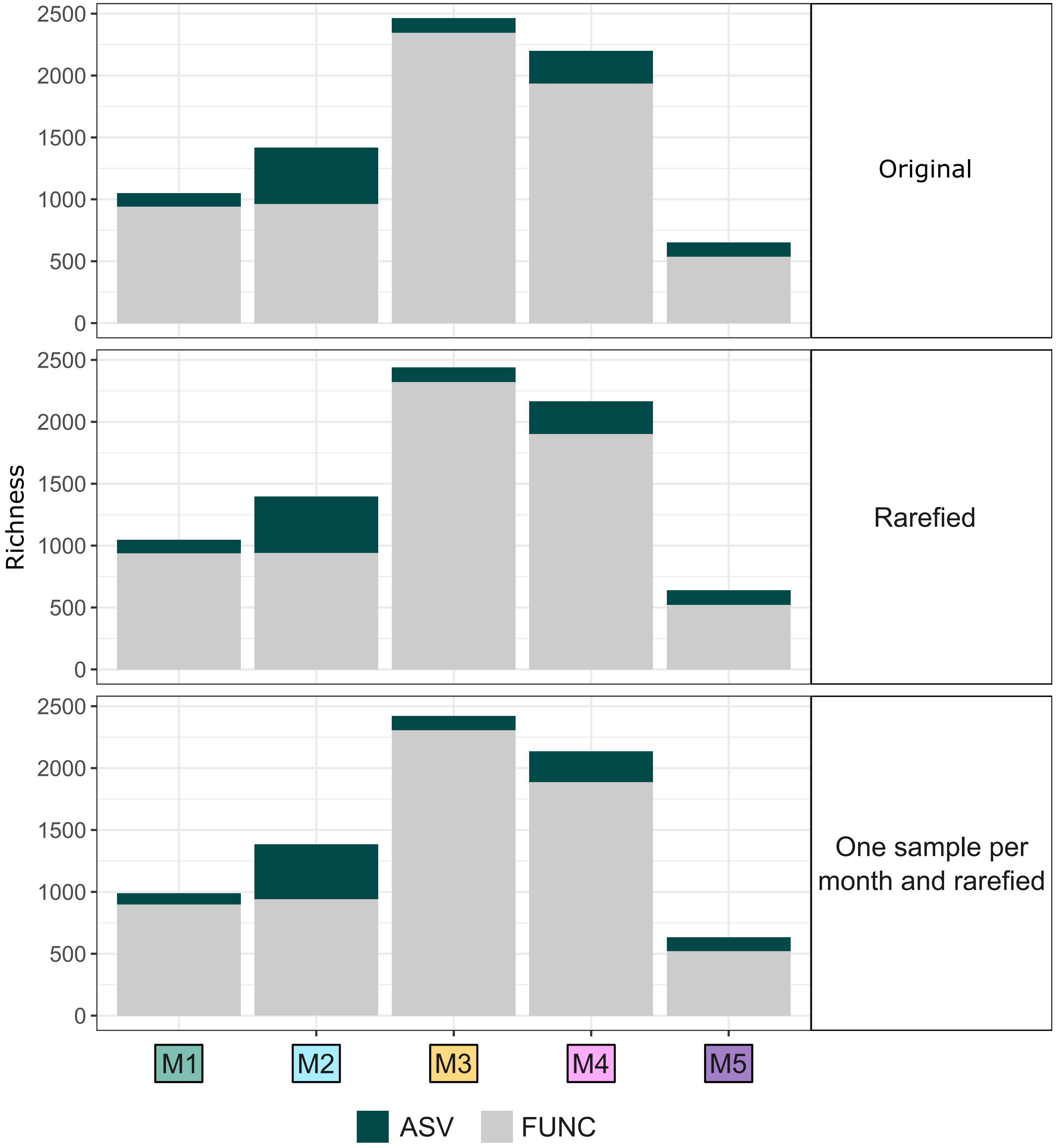

Supplementary Figure 2. Taxonomic and functional richness of modules. Richness was assessed based on the number of ASVs and functional clusters identified in each module. Todetermine whether richness was influenced by differences in sequencing depth or thefrequency of sampling across seasonal periods, we also assessed richness after rarefying the ASV and functional cluster counts and also after subsampling to one sample per month and then rarefying.

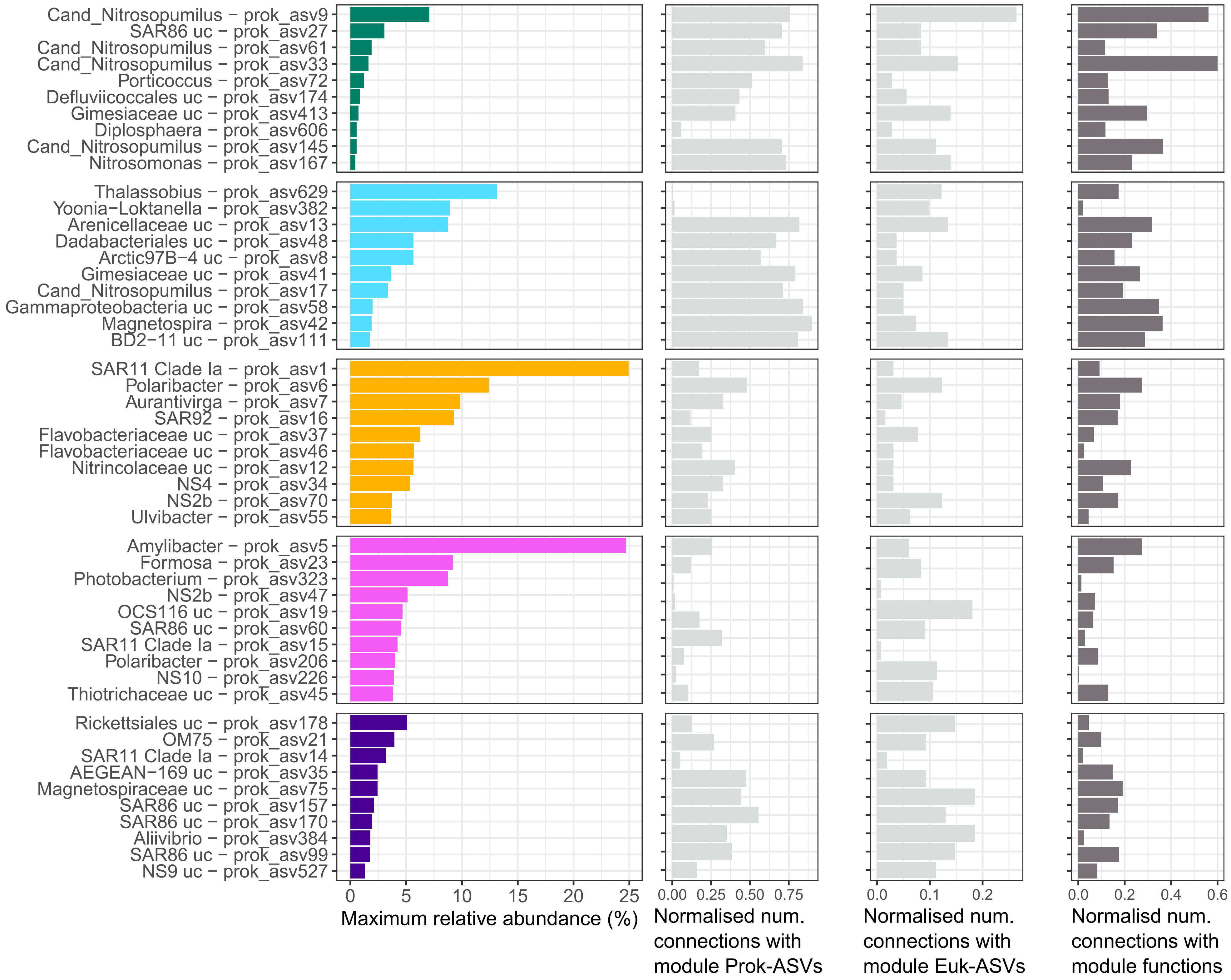

Supplementary Figure 3. Most abundant prokaryotic ASVs in modules and their network connections. Ten most abundant prokaryotic ASVs identified in each module, based on maximum relative abundance reached, along with the number of network connections to prokaryotic and microeukaryotic ASVs and functional clusters within the same module, after normalising by the total number of nodes in each module.

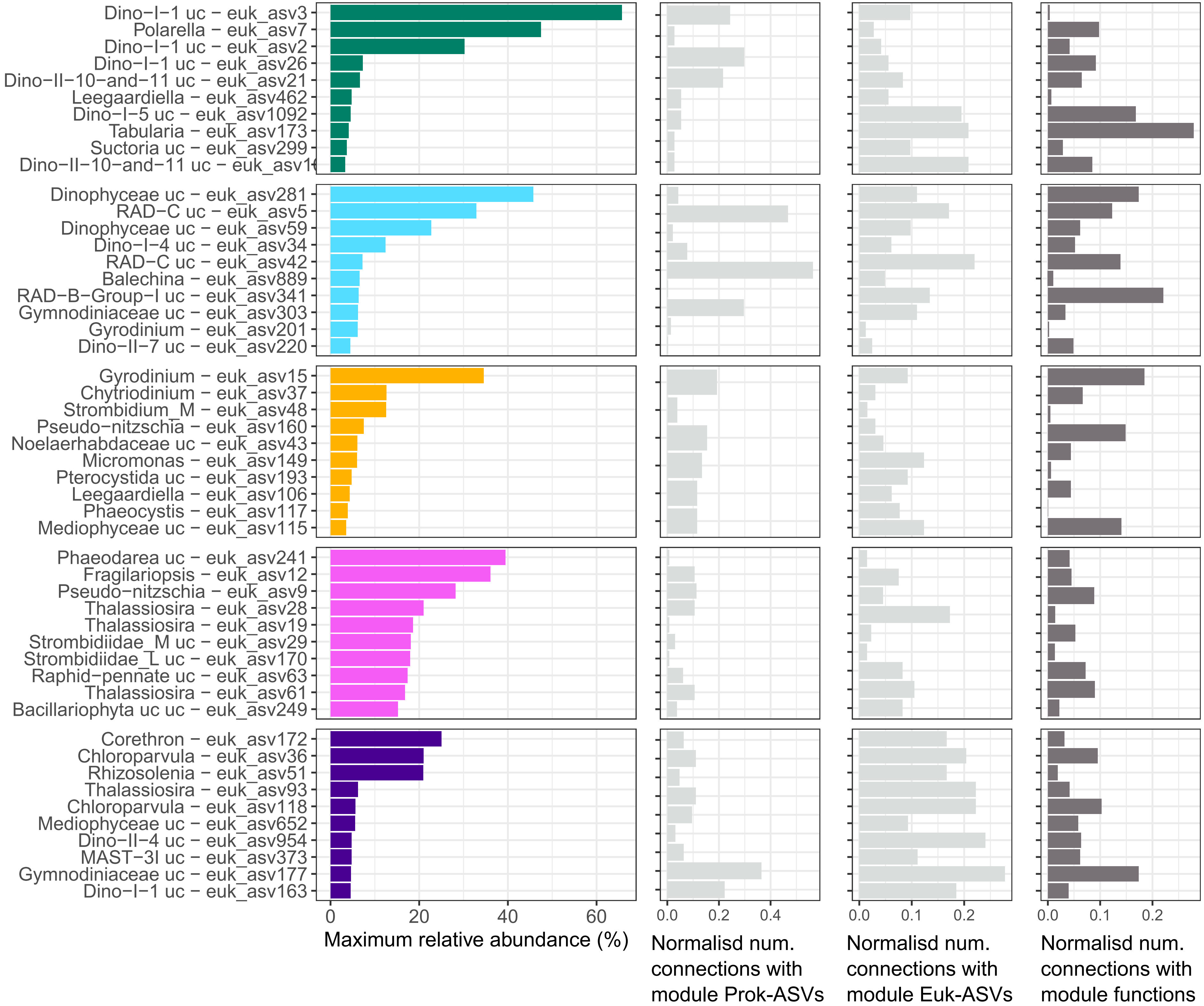

Supplementary Figure 4. Most abundant microeukaryotic ASVs in modules and their network connections. Ten most abundant microeukaryotic ASVs identified in each module, based on maximum relative abundance reached, along with the number of network connections to prokaryotic and microeukaryotic ASVs and functional clusters within the same module, after normalising by the total number of nodes in each module.

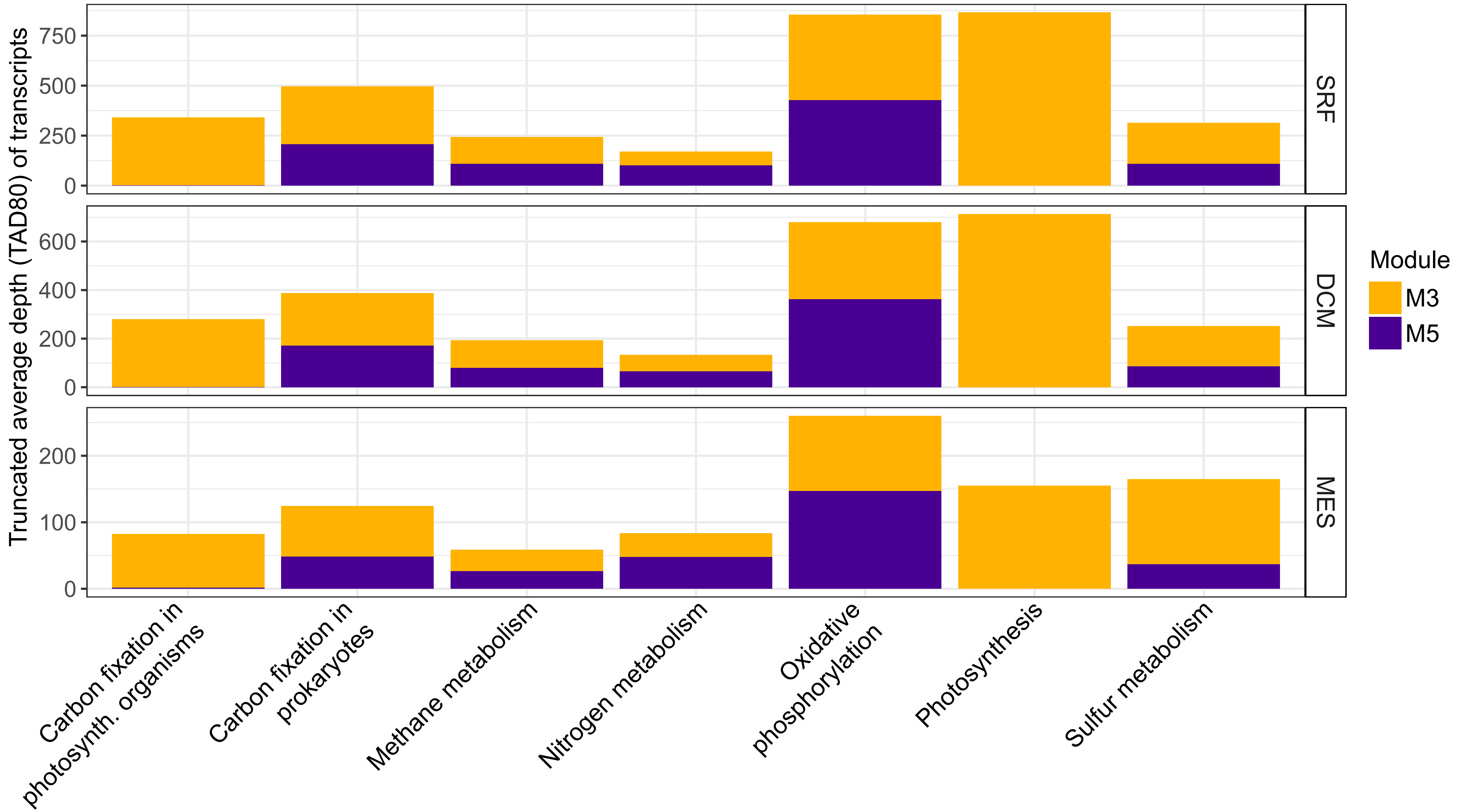

Supplementary Figure 5. Mean transcript depth of energy metabolism functions in module M3 and M5 in Arctic Ocean samples. Transcription of module functions was determined by read recruitment from metatranscriptomic data generated from surface, deep-chlorophyll maximum and mesopelagic depth layers of the Arctic Ocean during the Tara Ocean expedition. For each module function, the 80% truncated average depth (TAD80) of transcripts was calculated. The mean TAD80 values were determined for each function in each depth layer. For functions associated with KEGG energy metabolism modules, the mean TAD80 values in each depth layer were summed, providing a mean transcription level for each metabolism. Illustrated in this figure are the mean values for modules M3 and M5, as they were the most highly transcribed of all modules.
