# Supplementary material for: Seasonal recurrence and modular assembly of an Arctic pelagic marine microbiome
Source: Nat Commun. 2025 Feb 3;16:1326. doi: 10.1038/s41467-025-56203-3 (PMC11790911; doi:10.1038/s41467-025-56203-3)
Supplement: Supplementary file 3 — Description of Additional Supplementary Files [file 41467_2025_56203_MOESM3_ESM.pdf]

Description of Additional Supplementary Files

File Name: Supplementary Data 1
Description: Information on the collection of samples through autonomous sampling devices and the environmental conditions during sampling. Temperature, salinity and oxygen measurements were obtained from Seabird SBE37-ODO CTD sensors attached to the
moorings. Photosynthetically active radiation values were obtained from AQUA-MODIS satellite data (Level-3 mapped; SeaWiFS, NASA). Mixed layer depth was determined by examining temperate and salinity values from CTDs positioned at two different depths on the mooring.

File Name: Supplementary Data 2
Description: ENA accessions for raw 16S and 18S amplicon and PacBio HiFi metagenome data.

File Name: Supplementary Data 3
Description: Taxonomic profile of prokaryotic ASVs. ASVs were created using DADA2 and assigned a taxonomy from the SILVA SSU REF138 NR99 database. Values provided are raw count values.

File Name: Supplementary Data 4
Description: Taxonomic profile of microeukaryotic ASVs. ASVs were created using DADA2 and assigned a taxonomy from the SILVA SSU REF138 NR99 database. Values provided are raw count values.

File Name: Supplementary Data 5
Description: Alpha diversity metrics of prokaryotic and microeukaryotic communities. The three diversity metrics were calculated using the vegan package in R after 100 iterations of rarefying the ASV Count data . The presented values represent the mean and the standard deviation (SD) from the 100 calculations.

File Name: Supplementary Data 6
Description: Results from Pearson's correlations between alpha diversity metrics and measured environmental variables. Statistically significant ($p < 0.05$) Pearson's correlation results after applying multiple testing correction using the Benjamini-Hochberg approach (p_value_adjusted).

File Name: Supplementary Data 7
Description: Convex hull areas from NMDS ordinations. NMDS ordinations were computed from Bray-Curtis dissimilarities of prokaryotic and microeukaryotic hellinger-transformed taxonomic profiles. Convex hulls were generated for samples based on the month the sample was collected. The area of the convex hulls were determined using the areapl function of the splancs package in R.

File Name: Supplementary Data 8
Description: Composition, quality and taxonomy of metagenome-assembled genomes. Completeness and contamination values were derived from CheckM v1 while taxonomy was determined using the GTDB r220 database.

File Name: Supplementary Data 9
Description: Results from Pearson's correlations between the abundance of module components and measured environmental variables. Statistically significant ($p < 0.05$) Pearson's correlation results after applying multiple testing correction using the Benjamini-Hochberg approach (p_value_adjusted).
